# First-Year College Student Life Experiences during COVID-19 in South Korea

**DOI:** 10.3390/ijerph18189895

**Published:** 2021-09-20

**Authors:** Mikyung Jun, Songyi Lee, Taeeun Shim

**Affiliations:** 1 Department of Home Economics Education, College of Education, Dongguk University, Seoul 04620, Korea; mkjun@dongguk.edu; 2 Dharma College, Dongguk University, Seoul 04620, Korea; 3 Competency Development Center, Dongguk University, Seoul 04620, Korea; shim2593@hanmail.net

**Keywords:** COVID-19, first-year college students, experience, adaptation, growth

## Abstract

The purpose of this study is to examine the first-year students’ experience in college during the COVID-19 pandemic to provide a better understanding of their daily life. Using inductive content analysis, this study examined the characteristics and experiences of students who started college during the COVID-19 period in South Korea. We analyzed 623 pieces of content, using data presented by a total of 81 study subjects. From this analysis, we derived 22 primary keywords, which we divided into eight categories, and then reclassified into three general topics: self-awareness (i.e., self-reflection), activities (i.e., engagement in activities), and resources (i.e., creating relationships or producing results). The results showed that, although first-year college students experienced difficulties in adapting to the COVID-19 situation, they tried to cope with them. Our findings shed light on the experiences of college students who experienced psychological problems during the COVID-19 pandemic and overcame related challenges.

## 1. Introduction

The COVID-19 pandemic is predicted to eventually transition into an endemic situation [1]. As in other countries, the so-called “Corona semester” and the general chaos of COVID-19 have caused mental health difficulties for students, faculty, and staff in South Korea’s schools [2]. However, this was not the first time an infectious disease produced such chaos in South Korea. Indeed, most universities initiated an early school vacation during the spread of MERS, which also caused confusion in academic management and led to the suspension of clinical training [3].

While COVID-19 proved more serious and long-lasting than these previous experiences, the public responses have progressed rationally and efficiently. The new normal emerged as people turned the crisis into an opportunity. In particular, schools adapted surprisingly quickly to new education methods [4]. COVID-19 forced all teachers to implement online lectures, which most had previously avoided, leading to changes in educational methods [5]. The pandemic also forced teachers and learners to adopt methods that promoted much greater convenience, safety, and efficiency in certain areas. This became a standard, a “new normal”, in the education field. During the Corona semester, professors videotaped lessons that students could listen to as many times as they wanted. In addition, teachers and students carried out video discussions and presentations, conducted virtual experiments, and took examinations online. Students also experienced the convenience of technology [6,7,8]. After COVID-19, these experiences will provide both teachers and learners new educational options.

This study investigates the college life experiences of students, especially first-year students, who went through the “Corona semester”. In South Korea, the college entrance rate was 70.4% in 2020; 72.5% of high school graduates entered college, which is a higher-level school [9]. In general, South Korean high school students enter college at the age of 19 years; females spend four years in college and male students spend six-to-seven years, because of mandatory military service in their early adulthood. We focused on first-year college students in the COVID-19 context for the following three reasons.

To begin with, first-year college students have finished their adolescence. Positioned in the early developmental stage of adulthood, self-growth is one of the essential developmental tasks they face [10]. During this transitional period, when changes and various competitions occur, students must respond appropriately to the demands of college life, in order to succeed in their studies, interpersonal relationships, and emotional development [11]. Many students enter college without preparing for their studies [12], and 4 out of 100 first-year students suffer from suicidal impulses in South Korea, demonstrating the difficulty of adapting to this new environment [13]. 

In 2020, individuals entering universities as first-year college students had to adapt to university environments undergoing an unprecedented reorganization due to COVID-19. This coincided with a time of transition in their lifecycles. The first-year college students of 2020 experienced the triple distress of adapting to the life transition from adolescence to early adulthood, to the transition from high school to college, and to the unprecedented university environment. This study investigates their experiences in this context and the meaning they gave to these experiences.

Second, the entrance exam culture in South Korea means that students entering college have already succeeded, despite very serious competition. Even given the present population situation, where the ultra-low birth rate means that university enrollment quotas exceed the number of high school students, admission to a “good” university is still vital to Koreans [14]. On the day of the government-sponsored college scholastic ability test, office workers start later in the day to help reduce the traffic congestion experienced by examinees, and the stock market opens late. In addition, when the English listening test is in progress, flights near more than 1000 examination sites across the country are completely controlled [15]. These measures are meant to ensure the convenience and fairness of the entrance exam environment. As such, entrance into university represents liberation from the evaluation of high school life records, which, from the students’ perspective, determine their success or failure on university entrance exams and in private education, such as the programs provided by private educational institutes and private after-school tutoring. 

Teachers and parents mention “the romance of the campus” as one means of encouraging students and children to endure the entrance exam hell. High school students make lists of things they can enjoy in college life. For example, they identify flexible university timetables, OT (Orientation) and MT (Membership Training) in March, festivals in May, long summer and winter vacations, and shopping with the money they had earned from the part-time jobs they held while enduring the difficulties of high school life. Thus, the first year of college typically functions as a sort of compensation for the first-year students who survive the challenging entrance exam. However, the COVID-19 pandemic made it impossible for students to enjoy the romance of the campus, thereby aggravating their psychological difficulties. According to Hawley et al. [16], college students in seven countries—the USA, the Netherlands, Ireland, South Korea, China, Malaysia, and Taiwan—worry about education, safety, mental health, employment stability/finances, uncertainty about the future, and relationships in the COVID-19 era. This study concretely examined the meanings the first-year college of students of 2020 gave to their college lives, which are developing in completely different ways than they expected while preparing for the entrance exams.

Third, this study recognizes that people in society regard the students who started college in 2020 as the most unfortunate students in the world because of the COVID-19 pandemic. The public believes that these students did not receive proper congratulations for their high school graduation or college entrance because they did not participate in normal high school graduation or university entrance ceremonies. Indeed, the term “the cursed class of 2020” has become a common means of expressing pity for the first-year college students of 2020 [17]. This raises an important question: how have the members of “the cursed class of 2020” accepted their fate as first-year college students of 2020? 

Universities provide students time and space to accumulate various experiences, observe new phenomena, and participate in new things that shape their lives. The first-year college students of 2020 faced the vital task of adapting to college life during the COVID-19 pandemic while transitioning to early adulthood. The process of adjusting to college life involves “active exchanges” between students and the university environment [18]. First-year students must adapt to pursuing a major course of study while building new interpersonal relationships with professors, friends, seniors, juniors, etc. [19]. The psychological characteristics of first-year students, related to their adaptation to college life, are intertwined with their ego identities, self-esteem, self-efficacy, and self-leadership. Failure to adapt effectively can produce destructive and painful results [1].

Therefore, this study examines the college life experiences of first-year college students transitioning to early adulthood in the unprecedented university environment caused by COVID-19 in 2020. To achieve our aims, we seek to answer the following research questions:

1. How have first-year college students experienced the COVID-19 period?
2. What meanings have first-year college students given to their life experiences during the COVID-19 period?

## 2. Materials and Methods

The data collection of this study was conducted in two stages. First, an open-ended questionnaire, related to the research topic, was analyzed to investigate their college life experiences during COVID-19. A focus group interview was then conducted to pursue in-depth content of their daily life. This process makes it possible to understand their life in college, broadly and deeply.

First, this study analyzed the college life experiences of students taking liberal arts courses. We used an open-ended questionnaire based on inductive content analysis, a qualitative research method. Researchers generally apply inductive content analysis to unstructured sentences to group data through a process that includes open coding, category creation, and abstraction. Researchers code the data, focusing on the grouped sentences as concepts, categories, or topics. Then, they combine the codes with other open codes that include similar content, thereby combining subordinate concepts, categories, and themes. The coding outcomes become the basis for reporting the results of the inductive content analysis [20]. This method has the advantage of enabling a clear interpretation of first-year college students’ experiences, without impairing researchers’ intentions [21].

Therefore, we used inductive content analysis to organize student interview data for open coding in this study and created topic categories to topicalize and abstract freshmen students’ college life experiences [22].

Second, focus group interviews were conducted. To select the participants, an e-mail was sent to first-year students taking liberal arts courses to ask if they were willing to participate in the study, and among the students who expressed their intention to participate, five first-year students studying in various departments were selected.

### 2.1. Study Subjects

#### 2.1.1. Documented Materials

This study examined how first-year college students experienced college life during the COVID-19 period. We asked 91 first-year students taking liberal arts courses to fill out an open-ended questionnaire focusing on the general question: “How was your college life experience during the last one year?” After excluding 10 international students from our survey, due to their culturally biased experiences, we analyzed a total of 81 questionnaires. The study included 32 males and 49 females. The majors of the 81 respondents were as follows:

- 11 in law
- 8 in English
- 7 in business
- 7 in physical semiconductors
- 5 in Chinese
- 5 in history education
- 5 in Japanese
- 5 in mathematics education
- 4 in geography education
- 3 in accounting
- 3 in Korean language education
- 3 in Korean literature and creative writing
- 3 in mathematics
- 3 in public administration
- 2 in history
- 2 in statistics
- 1 in chemistry
- 1 in education
- 1 in home economics
- 1 in media communication
- 1 in physical education

#### 2.1.2. Focus Group Interview (FGI)

To further investigate this study’s topic using the collected data, we conducted focus group interview (FGI) with five first-year college students from different colleges: business, humanities, life science and biotechnology, law, and natural sciences. We used WebEx for the interviews. We explained the study’s purpose to the subjects and obtained their informed consent. We compensated each interviewee 20,000 KRW for their participation. The interviews included in-depth questions, such as: “What experiences did you have as a first-year college student of 2020?” and “What changes did you feel while you were taking classes during the first and second semesters?” We recorded and transcribed the content of the student interviews.

### 2.2. Study Procedure

#### 2.2.1. Collection and Analysis of Documented Materials

This study collected documented materials on the college life experiences of 81 first-year students for the year 2020. The researchers separately read the responses of 81 first-year college students several times to become familiar with the answers, extracting and organizing sentences related to their experiences as first-years. They subsequently re-read the materials and tried to find important meanings as themes, trying to understand the participants’ experience in college during the COVID pandemic. The researchers repeatedly read the written materials and wrote as many titles as necessary to explain all their dimensions [23,24,25]. Then, they collected their titles, avoiding overlapped meanings. They identified and refined the titles, resulting in 623 open codes. 

In this stage, researchers freely created titles, and three researchers held separate meetings to consider what criteria they should use to classify the transcribed materials, focusing on the 623 codes. In these meetings, the two researchers classified the materials by grouping similar concepts, extracting 22 keywords for the groupings, through concrete discussion of what keywords to create. They then grouped the 22 materials by combining categories with similar or different purposes into broader, higher-order categories to reduce their number [23,26,27]. In this process, when the researchers disagreed, they repeatedly read the data, focusing on what the participants intended and revised the categorization repeatedly. If an agreement was still not made, the third researcher gave an opinion for coding to increase the validity. Finally, they grouped the 22 categories, in order to explain the subjects’ college life experiences, improve understanding, and create knowledge regarding the subject. 

After that, we categorized the 22 open-ended extracted words into eight content areas. First, we grouped some words into “College life without any compass”, focusing on content related to “Giving up on the college life they envisioned”, “Feeling helpless”, “Feeling lost”, “Feeling overwhelmed”, and “Feeling limited”. Second, we grouped some words into “Interaction (network)”, focusing on content related to “Interacting with classmates”, “Interacting with senior classmates”, and “Making new relationships in school”. Third, we categorized some words into “Stagnation”, focusing on content related to “Feeling like it is an extension of high school”, “Feeling bored with the repetitive lifestyle”, and “Staying at home all the time”. Fourth, we included a category for “Extracurricular activities” focusing on content related to “Engaging in club activities”, “Working at a part-time job”, and “Being involved in student council” during college life. Fifth, we sorted some words into “Self-reflection”, focusing on content related to positive and negative introspective insights. Sixth, we combined some words into “Curriculum activities”, focusing on content related to “Taking online courses” and “Taking courses” for college classes. Seventh, we placed some words in a “Competency” category, focusing on content related to “Feeling a sense of accomplishment” and “Having an increased awareness of better time management”. Finally, in the eighth category, we grouped words into “Adaptation”, focusing on content related to “Setting new goals” and “Adjusting to the given situation”.

In the concluding topic extraction stage, we identified the final categories as self-awareness (i.e., insight into oneself), activities to intervene in activities, and drawing on resources to establish relationships or produce results. We then calculated the frequencies and percentages of the 623 pieces of material, used in the content analysis that we referred to, as we interpreted the study findings.

#### 2.2.2. FGI Data Analysis

We recorded, transcribed, and analyzed the content of the interview with five first-year college students and described the results to concretize meanings by connecting the content with the analytic results. In other words, we used the results to provide in-depth explanations of the meanings of individual topic classifications.

## 3. Results

### 3.1. Topic Classification

We obtained 623 pieces of content about life experiences from first-year college students and classified them into 22 primary topics, categorized these detailed areas into eight secondary topics and further classified these secondary topics into superordinate concepts, such as self-awareness (i.e., self-reflection) (49.76%), engagement in activities (i.e., to engage in activities) (33.07%), and resources (making relationships or achieving results) (17.17%). All the topic classification about life experience from first-year college students is displayed in Table 1.

### 3.2. Topic Findings

#### 3.2.1. Self-Awareness

Our analysis of first-year college student life experiences identified the topic “life without any compass” in 26.16% of the content. “Life without any compass” refers to frustrating experiences due to the cancellation of MT, clubs, festivals, etc., that first-year college students expected to be part of college life. It also refers to the sense of helplessness that resulted from the limitations on external activities, the confusion stemming from the lack of guidelines for college life, and feeling overwhelmed by the responsibility of simultaneously undertaking college life and abiding by activity restrictions while desiring to engage. This content shows that students experienced frustration in unexpected situations. For example, student “A” (who participated in the interview) said:

*“If I go to school, I can discuss everything with my classmates and meet seniors for help, but since I could not go to school, I encountered difficulties in the process of registering for courses and attending lectures, the process of performing tasks, and the process of preparing for employment”.*

We found the topic of “self-reflection” in 11.88% of the content. This topic encompassed, feeling grateful while engaging in self-reflection, regarding increased personal time and positive self-perceptions, derived through the process of valuing even small things. It also included content related to introspection, regarding wasting time and not trying to grow. We found that first-year college students had time to reflect on themselves, whether positively or negatively, during a period that became relatively long. For instance, student “B” said:

*“I think I have only vaguely thought about my future and never seriously thought about it, but because I have had a lot of time to worry and because I looked for a lot of things I could do at home because of the non-face-to-face classes, I had opportunities to seriously think about the future regarding how to live and what values I should pursue in my life”.*

The topic “adaptation” occurred in 11.72% of the content. This topic encompassed content related to making and practically carrying out plans to adapt to, accept, and succeed in the new, unexpected environment. Through trial and error, first-year college students resolved to spend their time meaningfully, no matter what settings they encountered in the future. In addition, they appeared to try to identify what they could do and then took action to do it in an environment that they could not change. For example, student “C” said:

*“Since non-face-to-face classes are required, and situations, where social distancing is required have persisted because of Corona, I think I limited what I can do by myself. Therefore, I thought I was limited in doing meaningful things. Thus, I tried to participate in more varied activities online”.*

In addition, student “D” said:

*“I was embarrassed very much at first because my college life was so much different from what I had expected, but later I tried to engage in many meaningful activities that I could do at home while practicing social distancing. I challenged myself to take the TOEIC test, and I tried to study for various certificates”.*

During the COVID-19 pandemic, these first-year college students experienced disappointment because this period did not align with their life expectations; they sometimes felt that they had wasted their time. However, we found that the extra free time they received enabled them to gain insight into themselves and that they tried to figure out what they could do to adapt to the pandemic environment. The contents of self-awareness for reflection are displayed in Table 2.

#### 3.2.2. Activities

The topic “stagnation” appeared in 4.65% of the content. Stagnation refers to the feeling of suffocation some first-year college students felt because college life did not differ significantly from high school life and because non-face-to-face classes occupied most of their time. They experienced boredom because of the repetitiveness of their daily patterns. Respondents described being unable to have new experiences, due to the increased time at home. However, they also positively characterized the experience, pointing out that the increased interactions and conversations fostered greater intimacy between family members. For example, student “E” said:

*“My grandmother moved to Busan and lives there. But when I was in my third year of high school last year, my grandmother came to Seoul and took great care of me. Now I can go to Busan and repay her for taking care of me”.*

The topic of “extracurricular activities” arose in 8.35% of the content. This topic refers to first-year college students’ experiences of club activities, part-time jobs, and student council activities, within the limits allowed by society. Respondents expressed gratitude for being able to interact with people through such encounters and claimed that the experiences brought them a sense of accomplishment. For example, student “F” said:

*“I am currently working to promote student record screening as a notifier under the title “dreamer” for the admissions office. Therefore, I went to school quite a bit. I think meeting people in person has advantages over non-face-to-face meetings”.*

We found the topic “curriculum activities” in 20.06% of the content. First-year college students regarded the knowledge acquisition, assignments, exams, and team projects they carried out in college courses as meaningful. They highlighted both the frustration they experienced with the processes of non-face-to-face classes and the advantages of these processes. For example, student “G” said:

*“I think that non-face-to-face classes certainly have advantages and disadvantages. In terms of distance, in fact, I have thought that the time to go to school and come back home was quite meaningless. The degree of utilization of time became very high thanks to the current “untact” online classes, and I think this is an advantage of online classes. However, I feel sorry that I have been unable to feel the atmosphere of classes where classmates gather and study together because such an atmosphere is much different from the atmosphere of classes where each student studies alone”.*

Respondents claimed that non-face-to-face classes were a source of discomfort in their lives and that they were bored because they spent a lot of time at home. Nevertheless, taking university classes still gave them experiences reminiscent of normal university life. Also, some students experienced advantages in non-face-to-face classes. The contents of engagement in activities are displayed in Table 3.

#### 3.2.3. Resources 

The topic “interaction (network)” emerged in 8.51% of the content. This topic refers to the interactions students had with classmates, either offline or online, with seniors at conferences or seminars, and with new people through their schools, including students from different schools. We discovered that first-year college students interacted with others as much as the circumstances permitted and gave positive meanings to such interactions. For instance, student “G” said, “The last year was a year in which meeting with people felt very precious”. Similarly, student “E” said, “I was afraid to register for courses for the first time, but I was happy because my seniors helped me”. 

The topic “competency” arose in 8.67% of the content. This topic encompasses content related to the scholarships the first-year college students received because of their dedication to their studies, as well as the personal progress they made by managing themselves and effectively utilizing their extra free time (away from college). For example, student “G” said:

*“The best thing about non-face-to-face classes is the aspect of time management. Going to school on the subway in the morning is exhausting, but since I do not have to go to school, there are so many things I can do on my own. Therefore, I spent the last year more worthily meeting other people and doing other meaningful things”.*

Ultimately, first-year college students strived to develop within their given environments. They appeared to work hard to grow, despite the circumstantial challenges they faced. The contents of drawing on resources to create relationship or produce results are displayed in Table 4.

## 4. Discussion 

This study examined the college life experience of first-year college students in an environment changed by COVID-19. We classified the content related to their college life experiences into three broad categories: self-reflection, engagement in activities, and use of resources to create relationships or achieve results. The results are discussed in several ways (as follows).

To begin, first-year college students experienced disappointment in their college lives, which differed from what they had expected; they also experienced psychological difficulties adapting. According to a study that analyzed 1000 news articles from December 2019 to October 2020, the most prevalent keywords during this period were “psychology”, “COVID-19”, “blue”, and “anxiety”. The spread of COVID- 19 has had a huge impact not only on daily life but in many areas, such as socioeconomics [28]. In addition, the uncertainty and unpredictability of COVID-19 have threatened people’s physical and mental health and had especially strong emotional and cognitive impacts [29]. For example, one study found that about 25% of Chinese university students experienced anxiety symptoms due to the COVID-19 pandemic [30]. However, these psychological difficulties varied among individuals and across situations. 

Yang [31] demonstrated that first-year college students with below-average ego resilience experienced higher levels of depression. Other studies showed that emotions such as anxiety, restlessness, stuffiness, helplessness, anger, fear, confusion, and distrust (due to COVID-19) were higher than among those with below-average ego resilience than in those with above-average ego resilience and self-efficacy [32,33]. These results support this study, in that we found that college students faced psychological difficulties because of their status as first-year college students. They experienced a sense of loss, due to the lack of the meaningful experiences they had expected from their college lives. They had to go through their experiences helplessly and alone, with insufficient information about college life. In other words, the psychological difficulties experienced by the first-year college students included senses of loss, helplessness, and being overwhelmed, all while encountering the limitations imposed by a new environment without a support system. 

Jeon and Yune [34] found that first-year college students experienced intrinsic difficulties adapting to school. They showed “a sense of burden for freedom”, “their ego faced disappointment due to expectations”, and they experienced “confusing college classes”. That study’s first-year college student respondents, thus, resembled the first-year college students of 2020 of this study, in that they experienced psychological difficulties, but the content of those difficulties was different. Those who were anxious felt stuffy and were depressed; additionally, they were healed by people who positively accepted them, understood them empathically, and treated them truthfully [35,36]. Our results support this research, although it was more difficult for our respondents to adapt to college life in 2020. The first-years we studied had insufficient opportunities to meet those who could relieve their difficulties, due to the burdens of college classes and activity restrictions 

Second, first-year college students had opportunities to reflect on themselves during the difficult time of COVID-19. According to Wang, Chen, Lin, and Hong [37], college students’ self-reflection affects their positive thinking and academic motivation. In addition, research has revealed that self-reflection and insight are positively related to coping ability [38]. This means that this study’s first-year college students had sufficient time to think about their school lives; in the past, they had always felt that their obligations and responsibilities left them with insufficient time. Although students must fulfill various roles and duties to develop, they also need opportunities to think and reflect independently. Our findings mean that South Korean society has not allowed sufficient space and time for self-reflection during adolescence, and students need enough time for reflection.

Third, although first-year college students experienced restrictions on their activities and had boring college existences that resembled their high school lives during the pandemic, they tried to find and practice potential college activities. They also derived a sense of meaning from taking college classes. 

They tried to stay at home to be safe from the risk of infection. This context resembles that of a study conducted by Latiffani and Hasanah [39], where STMIK Royal Kisaran students canceled all events and stayed at home to abide by physical and social distancing requirements. However, staying at home is a greater barrier to student activity during the first year of college than in other developmental stages. College students are the healthiest they will be in their lives, but restrictions on their radii of action can create high stress [40,41]. In addition, if they avoid going out and stay home all the time out of fear of infection, the state of “self-isolation” they create is more likely to increase their stress than relieve their anxiety [42].

Students experienced boredom in the restricted environment, but they also expressed satisfaction with their interactions with their family members. Our findings differ in this regard from another study that examined the changes in the daily lives of families during COVID-19 [43], which indicated that stress factors that had not existed earlier increased in areas such as education and care. These divergent results and the fact that the subjects of this study were in a developmental stage where they could live independently suggests that their perceptions of shared time in daily family life varied according to their family members’ developmental stages. In this study, first-year college students found that the experience of a family bond, in the context of COVID-19, was highly meaningful. 

Fourth, first-year college students experienced the negative and positive aspects of online college classes and came to understand the meaning of college classes per se. According to a college student survey concerning the emergency distance learning experience due to COVID-19, Wi-Fi quality and finding a quiet place were important factors in taking classes. This new situation can tire students and lead them to become critical of classes [44]. According to Barnes [12], time management, teachers’ low expectations/inconsistency, and continual student needs interfered with academic achievement, and systemic technical problems hindered students’ academic motivation [45]. This study’s findings, regarding students’ disappointment about non-face-to-face classes, align with those of previous studies. However, the respondents had positive views of the reduced time and cost associated with online classes. 

We also found that learning, doing homework, and taking exams in college were fun and meaningful, despite the non-face-to-face settings of the classes. According to Thomas [46], the first-year college students were more active in online classes than students in other grades. This finding suggests that students who had not experienced face-to-face classes at college adapted to the given environment because they had no basis for comparison. Many of these students expect that even when COVID-19 is over, online non-face-to-face classes will continue, depending on the situation. According to Fung and Lam [47], the online learning experience of the COVID-19 era could become the new norm in the emerging digital society and make a positive difference. The positive attitudes toward online classes of the freshmen in this study could reflect this move toward digital learning. Kang et al. [48] developed a service design that could solve the difficulties experienced by first-year college students—a “digital curation calendar”. The calendar could function within a smartphone application, to develop in-school curricular/extracurricular schedules that fit individual students’ schedules and fields of interest. These measures can help new students adapt to school in the post-COVID-19 era. 

Fifth, the first-year college students regarded exchanges with people in their limited environments as meaningful. They managed themselves and worked to produce even small results. Social support plays a significant role in the adaptation of college students [49]. Our study found that students felt joy and experienced a sense of their identities as college students in their interactions with new people, including their peers, seniors, and school personnel. 

Although first-year college students experienced psychological difficulties during the COVID-19 pandemic, they endeavored to adapt and develop themselves. They had to adjust their attitudes and behaviors, while accepting the changes resulting from COVID-19. To effectively adapt, they had to engage in challenging new behaviors and use strategies they had not used in the past. This involved a multifaceted coping process in the cognitive, psychological, and technological realms, and it appears that this progression can result in personal growth. Self-growth refers to how one becomes the person he or she is; it involves the ability to realistically understand oneself as one is, accept oneself as one is, and open oneself to others [50]. In short, the results of this study indicate that while students experienced psychological difficulties during the COVID-19 pandemic, they continued to grow. 

## 5. Limitations

First, although the character and intensity of COVID-19 have varied, depending on one’s family and social context, this study treated “COVID-19” as an exogenous variable. 

Second, we did not consider students’ personal characteristics. College life experiences and how students assign meaning to these experiences may vary, depending on the students’ majors. Other variables include whether students crammed to repeat the college entrance exam, whether they had experienced enrollment in other colleges, their gender, etc. We did not consider any of these personal characteristics in our study.

Third, the term “college life” in this study refers to life at universities where classes and extracurricular activities usually occur face-to-face. Therefore, this study does not explain the experiences of first-year college students in universities where online classes predominate, such as the broadcasting university. 

Lastly, even though we tried FGI to understand the first-year students’ college life deeply, we still focused on the written questionnaire data in this investigation, as we needed to begin to develop the parameters of further qualitative study.

## 6. Conclusions

This study examined the experiences of the first-year college students of 2020 and the meanings they assigned to their experiences in a college environment impacted by COVID-19. We analyzed the responses of 81 students to open-ended questionnaires, as well as the results of FGI with five students, and drew the following conclusions. First-year college students experienced a sense of loss because COVID-19 prevented them from having the college lives they expected. They suffered from helplessness, due to a lack of information about college life. However, at the same time, they turned their eyes to the positive aspects of non-face-to-face college classes. They re-evaluated the environment created by COVID-19 by exerting cognitive flexibility, turning it into an opportunity to reflect on themselves. The first-year college students who participated in this study tried to solve their problems with adapting to COVID-19 era college life by converting them into easily manageable issues. When they failed in this strategy, they tried to govern themselves through in-depth self-reflection. In sum, we found that, although first-year college students experienced difficulties in adapting to the COVID-19 situation, the process of coping with these difficulties enabled them to continue growing.

## Figures and Tables

**Table 1:** Topic classification.

Primary Topics	Secondary Topics	Superordinate Concepts
Giving up on the college life they envisioned (15.57%) Feeling helpless (4.33%) Feeling lost (3.21%) Feeling limited (2.41%) Feeling overwhelmed (0.64%)	Life without any compass (negative impact) (26.16%)	Self-awareness (49.76%)
The positive impact of extra time for self-reflection (7.06%) The negative impact of additional time for self-evaluations (reflection) (4.82%)	Self-reflection (11.88%)	
Adapting to the given situation (8.67%) Setting new goals (3.05%)	Adaptation (11.72%)	
Staying at home all the time (2.25%) Feeling like college life is an extension of high school (1.93%) Feeling bored with the repetitive lifestyle (0.48%)	Stagnation (4.65%)	Engagement in Activities (33.07%)
Engaging in club activities (4.49%) Being involved in student council (2.89%) Working at a part-time job (0.96%)	Extracurricular activities (8.35%)	
Taking online courses (11.24%) Taking courses (8.83%)	Curriculum activities (20.06%)	
Interacting with classmates (5.62%) Interacting with senior classmates (1.61%) Making new relationships in school (1.28%)	Interaction (8.51%)	Resources (17.17%)
Feeling a sense of accomplishment (7.54%) Having an increased awareness of better time management (1.12%)	Competency (8.67%)	

**Table 2:** Self-awareness for reflection (n = 310) (49.76%).

Content	Primary Topic (Detailed Area)	Secondary Topic (General Area)
My life as a college student was far from the so-called “campus life” that I had envisioned after finishing the preparation for the college scholastic ability test, which was hard. After the announcement of college acceptance, I looked forward to all the activities at the university, such as MT, OT, and freshman training, that my friends who entered college immediately after high school graduation boasted about. However, due to the sudden outbreak of COVID-19, all plans came to naught, and the semester began without face-to-face classes. I studied hard for three years in high school, bearing the romances of college life in my mind. These were small romances such as seeing celebrities at festivals, going on trips with classmates, or at least attending overlapping lectures with friends and taking classes together. However, these romances were shattered in a moment.	Giving up on the college life they envisioned (97) (15.57%)	Life with no compass at all (Negative impact) (163) (26.16%)
It seemed that I was repeating meaningless activities such as listening to lectures, sitting in the dormitory alone, doing homework, going to eat, and sleeping. As I spent more time at home without going out, my health naturally deteriorated. I lost a lot of muscle and felt mentally depressed. I thought I would have a lot of time for self-development because I would spend a lot of time at home, but I spent a lot of time just using my cellphone. Since I did not receive or even get a student ID or a department jacket, I often felt that I was not a college student but a jobless person in an ambiguous position.	Feeling helpless (27) (4.33%)	
I think “experience” is one of the most important things, but unlike the college life I envisioned during my school days, I experienced college life under many restrictions. Due to the corona situation, there were restrictions on various activities such as meeting new people and going out to play. Due to the Corona crisis, there were restrictions on various activities, such as meeting new people from new places, making friends, and going out to play.	Feeling limited (15) (2.41%)	
April and June were difficult periods with assignments, midterms, and final exams. Since I was experiencing college life and lectures as well as assignments and exams for the first time, I remember I experienced difficulties during these periods. Listening to classes sitting alone in my room without even going to see the campus was unmanageable sometimes.	Feeling overwhelmed (4) (0.64%)	
When the semester started, I had a very hard time. I wondered whether I was doing well in the non-face-to-face classes, and it was very difficult for me to face things in person with my own effort since I did not know any seniors. I was a little afraid as it seemed like I was progressing slower than others. I was afraid that while my classmates were doing their best and gradually achieving what they wanted, I might be lagging behind alone.	Feeling lost (20) (3.21%)	
College life for one year was a time to reestablish myself. The increase in the time given to me due to the nature of the non-face-to-face classes was very beneficial in that I had time to reflect on myself and to see whether what I was learning was what I really wanted and to figure out how to live in the future and plan my life. I had thought small things were nothing, but I knew that they were really precious, and I’m grateful.	The positive impact of additional time for self-reflection (44) (7.06%)	Self-reflection (74) (11.88%)
I am sorry that I don’t seem to have enjoyed my college life properly due to the corona situation, and rather I feel like my time was wasted in vain. I could not stand it because I was overwhelmed by the feeling that I had misled myself. Although I was vaguely aware that growth must be supported by self-understanding, I came to realize that I was quite insensitive to and ignorant about my own personality and emotions.	The negative impact of extra time for self-reflection (30) (4.82%)	
I took classes for the maximum number of credits I could get. Despite the concerns of those around me, I can say that I lived 2020 quite satisfactorily. It was a time when we all had to endure difficulties. I came to think that if I was going to spend my time watching YouTube anyway, I should do something productive; so, I started learning the piano and composing, which I always wanted to try at least once. I tried to reduce the amount of time I spend watching YouTube or Netflix through such productive activities, even if they were not studies. At first, I gave up the plan because it collapsed, but I made a plan again and acted accordingly	Adapting to the given situation (54) (8.67%)	Adaptation (73) (11.72%)
From now on, I think I should not be too greedy about my grades but should plan a schedule that takes my physical strength and leisure time into consideration so that I can focus on exams and studies. From the second year, I want to make a more systematic plan and work hard without being lazy so that I will not be disappointed by myself anymore. Thanks to non-face-to-face classes, I had relatively more time to spare, and I was able to have more time to think about my career and dreams. To make up for the one semester that I spent inattentively, I think I have to live more fiercely for the remaining three years.	Setting new goals (19) (3.05%)	

**Table 3:** Engagement in activities (n = 206) (33.07%).

Content	Primary Topic (Detailed Area)	Secondary Topic (General Area)
Because I rarely left the house due to the coronavirus, exchanges among family members increased. Therefore, I think the opportunities to respect, understand, and talk to each other increased so that we became more friendly to each other. My first year of university life seemed more futile than I expected because due to the coronavirus, I could not go to the campus so that I could hardly see my classmates and only stayed at home. I thought positively that since I have been taking classes at home, I had more time to spend with my family, had no need to go to the campus, and could have enough sleep.	Staying at home all the time (14) (2.25%)	Stagnation (29) (4.65%)
I do not even know the names and faces of my classmates in my department, and I do not know what the department room looks like. In addition, since I have been taking classes online, I do not think my college life was much different from my high school life. The first semester of the first year was not different from the fourth year of high school. It was difficult for me to feel college life in other areas than classes fully, and non-face-to-face lectures were unsatisfactory because I felt like I was listening to the Internet lectures I had been taking in high school for a year.	Feeling like college life is an extension of high school. (12) (1.93%)	
It seemed like I was repeating meaningless activities while I was sitting in the dormitory listening to lectures, doing homework, going to eat, sleeping, and so on. Since I did not participate in any club or the student council, had few friends at school, and felt stuffy, I spent time in lethargic and depressed states. Since I listened to lectures, did homework, and stayed only at home every day, my college life was not interesting, and I thought that my high school life was more interesting.	Feeling bored with the repetitive lifestyle (3) (0.48%)	
Because I was acting as a “dreamer,” which is a club people for the first-year students,” I interacted with friends from other departments I met as a dreamer. I was able to feel and experience the fact that the university is a place where I could meet different types of people and people from different regions. Of course, many activities were canceled due to COVID-19, but the few activities that were not canceled have become precious memories for me. It was nice to meet new people through club activities. Due to COVID-19, I could not carry out proper activities even once despite the fact that I joined a club. Therefore, I felt that the club was meaningless and ultimately withdrew. Although many things have been disrupted due to COVID-19, I gained at least one thing from my college life. I wanted to join a club without fail when I entered university. It was a broadcasting station. I was really happy when she became an announcer for an educational station by grandly passing the test to write a letter of self-introduction, written test, and interview.	Engaging in club activities (28) (4.49%)	Extracurricular activities (52) (8.35%)
I felt proud of the hard work I put in to help my classmates as a vice student representative in my department. I think I enjoyed working hard in this limited situation thanks to the student council activities, although I originally should have only taken online classes in Yeosu. I think I am fortunate because I went to school from time to time and became intimate with my classmates, students from other departments, and seniors while I was active in the student council.	Being involved in student council (18) (2.89%)	
Since March, I have been working at a simple job of teaching English to students at a private academy. This experience has enabled me to improve my English abilities and gain confidence. I found the new experiences I had working at the Central Library satisfying. Meeting with people while I visited the Korea Legal Aid Center for Family Relations and worked part-time became an opportunity for me to fulfill a personal dream.	Working at a part-time job (6) (0.96%)	
Everything such as lectures, practical training, interviews, freshman receptions, and events were carried out non-face-to-face. It was hard to accept that the non-face-to-face classes were our reward for studying hard for six years in middle and high school. Even after the coronavirus is over, I hope that online classes that reduce the burden of time and money will be actively used.	Taking online courses (70) (11.24%)	Curriculum activities (125) (20.06%)
Although I spent most of my daily life acquiring knowledge, exploring opinions and perspectives that I could not encounter in high school, and writing essays, this time helped me achieve inner growth. College life, that is, my every day, was really completely filled with getting up to sit at my desk and listen to classes on my laptop, submitting all the assignments the professor gave me in each class, and taking the midterm and final exams. The process of writing, presenting, and discussing the first academic presentation paper was really interesting and meaningful.	Taking courses (55) (8.83%)	

**Table 4:** Drawing on resources to create relationships or produce results (n = 107) (17.17%).

Content	Primary Topic (Detailed Area)	Secondary Topic (General Area)
Although I was unable to enjoy the first-year college life I wanted and experienced many difficulties, I think I escaped the worst situation because I became intimate with my classmates. While talking with classmates from various regions, I certainly told different stories from those I told when I was in high school. Because it was difficult to go outside due to the corona situation, I met with my classmates on WEBEX to have a good time while preparing for tests, playing games, and having conversations. When I received the notice that I should not go to the campus but should take non-face-to-face classes at the beginning of the semester, I agonized a lot about how to make friends, but I was very happy because I was able to make friends and felt that I was a college student while looking at my classmates.	Interacting with classmates (35) (5.62%)	Interaction (53) (8.51%)
Seniors made a schedule for me using KakaoTalk. Without their help, I would have created the worst schedule. I became close with seniors, and they bought me food so that I could feel like a college student. Although the processes of studying and preparing for seminars in the conference were not always enjoyable, learning to overcome the difficulties I encountered in these processes and interacting with seniors was very helpful.	Interacting with senior classmates (10) (1.61%)	
It was an opportunity to meet people who have read and learned different things in different environments and to share ideas equally regardless of age or gender. The thing I am most grateful for in my college life is human relationships. I met many good people, and I feel that I have found a more leisurely life than when I was in high school.	Making new relationships in school (8) (1.28%)	
I received great rewards in the form of good grades and scholarships. By living according to the planning I developed, I gained self-management abilities that enable me to succeed in my life. This gave me a sense of accomplishment. I started the second semester determined not to blame the current situation but to do my best to carry out small plans. For example, rather than starting an assignment 1–2 days before submitting it, I started in advance and got satisfaction by making small plans for specific periods and giving myself rewards when I finished assignments in time.	Feeling a sense of accomplishment (47) (7.54%)	Competency (54) (8.67%)
I used the extra time from the non-face-to-face classes to manage myself. It is a shame to waste my life doing nothing. Therefore, I tried to do everything I could. After meaninglessly a lot of time, I felt skeptical about my unproductive life. Therefore, I started trying to do productive things. For example, instead of playing on my cellphone, I went to a guitar academy and started learning electric guitar, which I had wanted to learn for a long time. I also tried various activities that can be done at home, such as cooking, French embroidery, font correction, and coloring. I had a lot of time. To fill it, I watched movie, read, and listened to music. I also kept a diary, which I had not done since elementary school, and it helped me a lot in my life.	Having an increased awareness of better time management (7) (1.12%)	

## Data Availability

Data from the study are available upon request.
